# Clinical and Biochemical Responses to SGLT2 (Sodium-Glucose Cotransporter-2) Inhibitors in Patients With Rheumatic Heart Disease and Heart Failure

**DOI:** 10.7759/cureus.116770

**Published:** 2026-09-23

**Authors:** Aman Asthana, Diganta Buragohain, Preethi Caleb Harris, Amish Shikoh, Amit Malviya, Animesh Mishra

**Affiliations:** 1 Cardiology, North Eastern Indira Gandhi Regional Institute of Health and Medical Sciences, Shillong, IND

**Keywords:** management of heart failure, new york heart association (nyha), n terminal pro bnp, rheumatic heart diseases, sodium-glucose cotransporter 2 (sglt-2) inhibitors

## Abstract

Background: Rheumatic heart disease (RHD) remains the third most common cause of heart failure in India, which bears the highest global burden of RHD. Left untreated or poorly managed, RHD culminates in heart failure, posing substantial challenges for clinical management. Despite advances in interventional management, the therapeutic landscape for heart failure in patients with RHD remains inadequately explored, particularly with respect to sodium-glucose cotransporter-2 (SGLT2) inhibitors.

Methods: This hospital-based study enrolled 90 patients with chronic RHD and New York Heart Association (NYHA) functional class II-IV heart failure. All patients received standard heart failure therapy, with SGLT2 inhibitors added according to the study protocol. Baseline evaluation included clinical assessment and measurement of serum N-terminal pro-B-type natriuretic peptide (NT-proBNP) levels. Patients were followed for three months, and changes in NYHA functional class and serum NT-proBNP levels were assessed.

Results: At three months following initiation of SGLT2 inhibitor therapy, significant improvements were observed in both clinical and biochemical parameters. The mean serum NT-proBNP level decreased significantly from 1,687.0 ± 897.8 pg/mL at baseline to 1,212.6 ± 606.6 pg/mL at follow-up (p < 0.001). Improvement in NYHA functional class was noted in 44 (48.9%) patients, while 41 (45.6%) patients remained clinically stable with no change in functional class. Five (5.6%) patients experienced worsening of NYHA functional class during follow-up. Overall, the change in NYHA functional class was statistically significant (p < 0.001).

Conclusion: Our findings suggest that the addition of SGLT2 inhibitors to standard heart failure therapy was associated with significant improvement in NYHA functional class and a significant reduction in NT-proBNP levels over three months.

## Introduction

India is labelled as the rheumatic heart disease (RHD) capital of the world, as it harbours 40% of the global burden [1]. According to the Indian Heart Failure Registry, RHD is the third most common cause of heart failure in India [2]. Left unrecognized or poorly managed, RHD often culminates in heart failure, posing substantial challenges for clinical management. Current management strategies focus on infection prevention and late-stage valve interventions. Despite advances in interventional management, the therapeutic landscape for heart failure in patients with RHD remains inadequately explored, particularly with respect to novel pharmacologic agents such as sodium-glucose cotransporter-2 (SGLT2) inhibitors [3].

SGLT2 inhibitors, in the last few years, have been extensively studied in heart failure patients, and based on large, randomized control trials, SGLT2 inhibitors are now integral part of guideline-directed medical therapy in heart failure patients across the spectrum of ejection fraction [4-7]. However, their efficacy in patients with RHD-related heart failure, a distinct population with unique pathophysiological characteristics, remains largely unexplored.

The rationale for investigating SGLT2 inhibitors in the setting of RHD-related heart failure stems from their unique pharmacodynamic properties, which may address several pathophysiological mechanisms underpinning this condition. Valvular dysfunction in RHD frequently results in elevated left atrial pressures, pulmonary congestion, and systemic venous hypertension, all of which contribute to the development of heart failure. By promoting natriuresis and reducing volume overload, SGLT2 inhibitors may alleviate symptoms of congestion, thereby improving functional status and quality of life. Additionally, the anti-inflammatory, anti-fibrotic properties and endothelial stabilization properties of these drugs may mitigate myocardial and valvular remodelling, potentially slowing the progression of RHD. Despite these theoretical advantages, the clinical applicability of SGLT2 inhibitors in RHD-related heart failure remains uncertain, as this population is underrepresented in major clinical trials. Existing evidence on SGLT2 inhibitors primarily originates from studies involving patients with ischemic or non-ischemic cardiomyopathy, with limited insights into their efficacy and safety in valvular heart disease. The aim of this study is to investigate the effect of SGLT2 inhibitors among patients with RHD with heart failure irrespective of the left ventricular ejection fraction.

## Materials and methods

Study design

This was a single-arm quasi-experimental study. The study was approved by the Institute Scientific Board and the Institutional Ethics Committee, North Eastern Indira Gandhi Regional Institute of Health and Medical Sciences (NEIGRIHMS), Shillong, India (approval no.: NEIGR/IEC/M12/T26/2023, dated: August 18, 2023). Consecutive patients aged >18 years with rheumatic heart disease (RHD) and chronic heart failure (New York Heart Association (NYHA) functional class II-IV) were eligible for the study [8]. Patients were diagnosed with heart failure based on 2021 European Society of Cardiology (ESC) heart failure criteria. The World Heart Federation echocardiographic criteria were used to establish the diagnosis of RHD [9]. The severity of native valvular lesions was assessed according to recommendations from the American Society of Echocardiography (ASE) [10,11]. Patients with known coronary artery disease, hypertrophic cardiomyopathy, constrictive pericarditis, restrictive cardiomyopathy, peripartum cardiomyopathy, infiltrative diseases causing cardiomyopathy, acute decompensated heart failure requiring IV diuretics or IV inotropes, previous percutaneous coronary intervention (PCI) or coronary artery bypass grafting (CABG) or other major cardiac intervention, chronic kidney disease (CKD) with eGFR <30 mL/min/1.73 m^2^, chronic liver disease and females with pregnancy or intended to be pregnant during the study period were excluded from the study. The patients who were already on SGLT2 inhibitors were excluded from the study.

Data collection

Detailed history was collected, and physical examination was done for all the eligible patients. At baseline, NYHA functional class was recorded for all participants. Investigations after enrolment include complete hemogram, renal function tests, liver function tests, serum electrolytes, lipid profile, haemoglobin A1c (HbA1c), random blood sugar and thyroid profile. A 12-lead surface ECG and detailed 2D echocardiography were done to assess the severity of valvular lesion, left and right ventricular function, and pulmonary artery pressure as per guidelines. Serum N-terminal pro-B-type natriuretic peptide (NT-proBNP) level was measured at baseline. NYHA functional class and serum NT-proBNP levels were evaluated at follow-up.

Visits and Follow-up

All patients included in the study received Dapagliflozin 10 mg or Empagliflozin 10 mg for three months after enrolment, along with pre-existing medications and reevaluated at three months.

Statistical analysis

Statistical analysis was performed using appropriate statistical software. Continuous variables were expressed as mean ± standard deviation (SD), while categorical variables were presented as frequencies and percentages. For comparison of serum NT-proBNP levels between baseline and three months, a paired t-test was used because the measurements were obtained from the same patients at two time points. The results were expressed as mean difference with corresponding test statistic and p-value. NYHA functional class at baseline and at three months was treated as a paired categorical ordinal variable. The change in the distribution of NYHA functional class between the two time points was assessed using the Stuart-Maxwell test for marginal homogeneity. A two-sided p-value of <0.05 was considered statistically significant. All statistical tests were two-sided, and a p-value of <0.05 was considered indicative of statistical significance.

## Results

Baseline demographic and clinical data

This study was conducted from July 2023 to June 2024. There was enrolment of 90 consecutive patients who fulfilled the inclusion criteria and had given informed consent. The majority of the participants were female (58 (64.4%)). The mean age of the participants was 51.35 ± 11 years.

A total of 58 (64.4%) patients had atrial fibrillation at baseline, and two patients had atrial flutter, while the remaining patients were in normal sinus rhythm. The majority of the patients were on heart failure medications in different combinations according to their clinical profile (Table 1). The majority of the patients (53%) were in NYHA functional class III, while 28.9% and 17.7% of patients were in NYHA functional class IV and class II, respectively, at the time of enrolment (Table 2). Baseline mean N-terminal pro-B-type natriuretic peptide (NT-pro-BNP) was 1,687 ± 897.8 (Table 3).

**Table 1:** Baseline Characteristics ACE: angiotensin-converting enzyme; ARNI: angiotensin receptor neprilysin inhibitor; MRA: mineralocorticoid receptor antagonist.

Variable (n = 90)		Number	Percentage
Age (years) Mean ± SD (51.35 ± 11)	< 30	11	12.22%
	31-50	39	43.33%
	51-70	40	44.44%
Gender	Male	32	35.56%
	Female	58	64.44%
Medications	Beta Blockers	74	82.22%
	ACE Inhibitors	26	28.89%
	Angiotensin Receptor Blockers	10	11.11%
	ARNI	15	16.67%
	DIGOXIN	33	36.67%
	MRA	74	82.22%
	Diuretic	76	84.44%
Rhythm	Atrial Fibrillation	58	64.44%
	Atrial Flutter	2	2.22%
	Normal Sinus Rhythm	30	33.33%
Ejection Fraction(%)			
	≥ 60%	53	58.89%
	40%-59%	23	25.56%
	<40%	14	15.56%

**Table 2:** Comparison of NYHA Class at Baseline and at 3 Months NYHA: New York Heart Association; n: number of patients. Stuart-Maxwell test was performed for statistical analysis. A p-value of <0.05 is taken as significant.

NYHA at Baseline	NYHA at 3 Months			
	Class I (n)	Class 2 (n)	Class 3 (n)	Class 4 (n)
Class 2 (n = 16)	2	11	3	0
Class 3 (n = 48)	0	19	27	2
Class 4 (n = 26)	0	2	21	3
Stuart-Maxwell test	χ² (3) = 30.81			
p-value	<0.001			

**Table 3:** Comparison of Mean Serum NT-proBNP Levels at Baseline and at 3 Months NT-proBNP: N-terminal pro-B-type natriuretic peptide. Paired t test was performed for statistical analysis. A p-value of <0.05 is taken as significant.

NT-proBNP	Mean NT-proBNP Levels (pg/mL) ± SD	
Baseline	1,687.04 ± 897.76	
3 Months	1,212.61 ± 606.63	
t value	7.138	
p-value	<0.001	

Baseline Echocardiographic Data

Baseline 2D echocardiography revealed that there was a high prevalence of mitral valve involvement, along with considerable involvement of aortic valve. Mitral valve involvement, either in combination of regurgitation and stenosis or stenosis alone, was seen in all patients. Among these patients, 99% had mitral stenosis and 66.7% had mitral regurgitation. A total of 38 (42.2%) patients in the study had aortic valve involvement. In this study, 53 (58.9%) had an ejection fraction of 60% or more, while 23 (25.6%) had an ejection fraction in the range of 40% to 59%. A total of 14 (15.6%) patients were having an ejection fraction of <40%. The mean pulmonary artery systolic pressure (PASP) at baseline was 63.4 ± 12.4 mmHg (Appendix 1).

Follow-up at three months

NYHA functional class and NT-proBNP level were assessed at three-month follow-up after starting SGLT-2 inhibitor. Fifty patients received dapagliflozin, and 40 patients received empagliflozin. The mean NT-proBNP level decreased from 1,687 ± 897.8 to 1,212.6 ± 606.6 (p < 0.001) (Table 3). Similarly, there was improvement of NYHA functional class in 44 patients at three-month follow-up. Forty-one patients had no change in NYHA functional class, whereas five patients experienced deterioration of NYHA functional class. The result was statistically significant (p < 0.001) (Table 2).

Safety Outcomes

No adverse events attributable to SGLT2 inhibitor therapy were reported during the three-month follow-up period. There were no documented episodes of symptomatic volume depletion, hypotension, worsening renal function, hypoglycaemia, genital or urinary tract infection, diabetic ketoacidosis, or treatment discontinuation due to adverse events.

## Discussion

This single-centre study evaluated the short-term effects of SGLT2 inhibitor therapy in patients with rheumatic heart disease (RHD) and heart failure across different ranges of left ventricular (LV) systolic function. The principal findings were a significant reduction in serum NT-proBNP levels and a significant change in NYHA functional class at three months following initiation of SGLT2 inhibitor therapy. These findings suggest that SGLT2 inhibition may provide symptomatic and biomarker benefits in patients with RHD-associated heart failure, a population that has been relatively underrepresented in major heart failure trials.

Comparison with previous studies

SGLT2 inhibitors have emerged as an important component of contemporary heart failure therapy. Randomized controlled trials have demonstrated reductions in heart failure-related events and improvements in symptoms across patients with reduced and preserved LV ejection fraction [5,7]. However, patients with rheumatic valvular heart disease have not been specifically represented in most of these landmark trials. This is clinically relevant because the pathophysiology of heart failure in RHD differs from that of many other forms of heart failure, with valvular obstruction and regurgitation, pulmonary hypertension, atrial arrhythmias, altered ventricular loading conditions, chronic inflammation and myocardial contributing to the clinical syndrome.

RHD remains an important cause of cardiovascular morbidity and mortality in low- and middle-income countries. In the Indian National Heart Failure Registry, RHD was reported in approximately 5.9% of patients with heart failure. Despite this substantial burden, evidence regarding the use of newer heart failure therapies, including SGLT2 inhibitors, specifically in RHD remains limited. The present study therefore provides preliminary real-world evidence regarding the potential role of SGLT2 inhibitors in this population.

Baseline characteristics and disease severity

The predominance of female patients in our cohort of 58 patients (64.4%) is consistent with the recognized epidemiology of RHD and with previous studies reporting a greater proportion of women among patients with rheumatic valvular disease [12]. The relatively advanced functional status of our study population was notable, with 48 (53.3%) patients in NYHA class III and 26 (28.9%) in class IV at baseline. This distribution differs considerably from the cohort described by Butt et al., in which most patients with non-ischemic heart failure were in NYHA class II and only a small proportion were in class IV [13]. Differences in disease aetiology, referral patterns, socioeconomic characteristics, duration of illness, and access to healthcare may account for this variation.

The high prevalence of atrial arrhythmias in our study is another important finding. Atrial fibrillation or atrial flutter was present in 60 (66.7%) patients. A meta-analysis involving 83 studies reported a pooled prevalence of atrial fibrillation of approximately 32.8% in patients with RHD, with substantial geographical and socioeconomic heterogeneity [14]. The higher prevalence in our study may reflect the advanced and chronic nature of rheumatic valvular disease in the study population.

Mitral valve disease was particularly prominent in our population, with mitral stenosis present in 89 (98.9%) patients and mitral regurgitation in 60 (66.7%). Aortic valve involvement was also observed in 38 (42.2%) patients. These findings emphasize the complex hemodynamic substrate underlying heart failure in RHD and distinguish this population from patients with predominantly myocardial forms of heart failure.

Effect on NT-proBNP

The mean baseline NT-proBNP concentration was 1,687.04 ± 897.76 pg/mL, reflecting substantial cardiac hemodynamic stress in this cohort. This value was broadly comparable to baseline NT-proBNP concentrations reported in the DAPA-HF trial [5]. Following three months of SGLT2 inhibitor therapy, the mean NT-proBNP level decreased significantly to 1,212.61 ± 606.63 pg/mL (p < 0.001), representing an approximately 28% reduction from baseline. This finding is consistent with the direction of change reported in major SGLT2 inhibitor trials. DAPA-HF demonstrated a significant reduction in NT-proBNP with dapagliflozin, while reductions in natriuretic peptide concentrations have also been observed in the EMPEROR-Reduced and EMPEROR-Preserved trials [6,7].

The reduction in NT-proBNP observed in the present study is clinically relevant because natriuretic peptide concentrations reflect myocardial wall stress and are associated with disease severity and prognosis in heart failure. Although the observational design does not allow a causal relationship to be established, the concordant improvement in NT-proBNP and functional status provides supportive evidence of a potential beneficial effect of SGLT2 inhibition in patients with RHD-associated heart failure.

Effect on functional status

A significant change in NYHA functional class was observed between baseline and three months following initiation of SGLT2 inhibitor therapy. This finding is consistent with the symptomatic benefits reported in major randomized trials of SGLT2 inhibitors. In DAPA-HF, dapagliflozin was associated with a significant improvement in NYHA functional class compared with placebo [5]. Similarly, EMPEROR-Reduced demonstrated a greater likelihood of improvement in NYHA functional status among patients treated with empagliflozin [6]. In patients with heart failure with preserved ejection fraction, EMPEROR-Preserved also demonstrated improvements in symptoms and health-related quality of life with empagliflozin [7].

The improvement observed in our study is therefore biologically and clinically plausible. SGLT2 inhibitors may exert beneficial effects through mechanisms extending beyond their glucose-lowering action, including natriuresis and osmotic diuresis, reduction in interstitial congestion, improvement in ventricular loading conditions, and favourable metabolic and renal effects. These mechanisms may be particularly relevant in RHD, where chronic volume and pressure overload frequently coexist with pulmonary hypertension and right-left ventricular interaction. Nevertheless, the change in NYHA class should be interpreted cautiously because NYHA functional classification is subjective and may be influenced by comorbidities, concomitant medications, and changes in clinical status unrelated to SGLT2 inhibitor therapy.

SGLT2 inhibitors across different LV ejection fractions

LV dysfunction in RHD may result from several mechanisms, including chronic pressure or volume overload caused by valvular lesions, impaired ventricular filling, ventricular interdependence, and potentially myocardial fibrosis related to recurrent rheumatic inflammation. Concomitant coronary artery disease or non-rheumatic cardiomyopathies may also contribute in individual patients.

Importantly, the present cohort included patients with preserved, mildly reduced, and reduced LV ejection fraction. A previous study by Das et al. from the same institution reported significant improvement in symptoms and NT-proBNP levels among patients with RHD and reduced LV ejection fraction treated with SGLT2 inhibitors [15]. The findings of the present study, together with these previous observations, provide preliminary evidence that SGLT2 inhibitors may have potential clinical benefits across different ranges of LV systolic function in RHD-associated heart failure.

However, this observation should not be interpreted as definitive evidence that the treatment effect is independent of LV ejection fraction. The present study was not designed or powered to formally evaluate treatment-effect modification according to ejection fraction. Larger studies with appropriate subgroup and interaction analyses are required to establish whether the benefits of SGLT2 inhibitors are consistent across different LV phenotypes in RHD.

Clinical implications

The findings of this study have potential clinical implications because patients with RHD and heart failure frequently have limited disease-specific therapeutic options. Management is primarily directed toward correction of the underlying valvular lesion, control of congestion and arrhythmias, anticoagulation when indicated, and optimization of conventional heart failure therapy according to the clinical phenotype.

The observed reduction in NT-proBNP and improvement in functional status suggest that SGLT2 inhibitors may have a role as an adjunctive therapy in appropriately selected patients with RHD-associated heart failure. Their potential benefits may be particularly relevant in settings where RHD remains prevalent and access to advanced heart failure therapies is limited. However, the findings should be regarded as hypothesis-generating rather than practice-changing because of the absence of a comparator group and the short follow-up duration.

Limitations

This study has several limitations. Firstly, it was a single-centre, single-arm study without a control group. Consequently, causality cannot be established, and the observed reductions in NT-proBNP and changes in NYHA functional class may have been influenced by regression to the mean, changes in concomitant therapy, natural variation in disease severity, or other unmeasured factors. Secondly, the follow-up period was limited to three months. Although this duration allowed assessment of short-term changes in symptoms and NT-proBNP, it was insufficient to evaluate long-term outcomes such as heart failure hospitalization, cardiovascular mortality, and all-cause mortality. Thirdly, the sample size was relatively small, limiting statistical power and the generalizability of the findings. The study was also conducted at a single centre, and the patient population may therefore not represent the broader spectrum of RHD encountered in different geographical and healthcare settings. NYHA functional class is a relatively subjective measure of functional status and may be influenced by factors other than cardiac function. Although the observed change was statistically significant, objective measures of exercise capacity and quality of life were not included.

Finally, evidence regarding SGLT2 inhibitor therapy specifically in RHD remains limited. Most evidence supporting these agents has been derived from trials involving predominantly non-rheumatic heart failure populations. Dedicated randomized controlled trials in patients with RHD are therefore required to determine the efficacy, safety, and long-term clinical benefits of SGLT2 inhibitors in this population.

Future directions

The findings of this study support the need for larger prospective multicentre studies evaluating SGLT2 inhibitors in RHD-associated heart failure. Future studies should include an appropriate comparator group, longer follow-up, and assessment of clinically meaningful outcomes such as heart failure hospitalization, cardiovascular mortality, quality of life, exercise capacity, renal outcomes, and changes in pulmonary pressures. Stratification according to LV ejection fraction, valvular lesion, severity of pulmonary hypertension, and presence of atrial fibrillation may also help identify patient groups most likely to benefit from SGLT2 inhibitor therapy.

## Conclusions

In this study of patients with RHD and heart failure, treatment with an SGLT2 inhibitor was associated with a significant reduction in serum NT-proBNP levels and a significant improvement in NYHA functional status over three months. These findings suggest a potential role for SGLT2 inhibitors as an adjunctive therapy in RHD-associated heart failure across different ranges of LV systolic function. However, given the limitations of the study design, these findings should be considered preliminary. Larger, prospective, multicentre randomized controlled studies are warranted to confirm the efficacy and long-term clinical benefits of SGLT2 inhibitors in patients with RHD.

## Appendices

Appendix 1 

**Table 4:** Baseline Echocardiographic Parameters

		Frequency	Percentage
Left Ventricle	Dilated	34	37.78%
	Normal	56	62.22%
Right Atrium	Dilated	55	61.11%
	Normal	35	38.89%
Right Ventricle	Dilated	26	28.89%
	Normal	64	71.11%
Mitral Valve	Thickened	89	98.89%
	Normal	1	1.11%
Aortic Valve	Thickened	38	42.22%
	Normal	52	57.78%
Tricuspid Valve	Thickened	6	6.67%
	Normal	84	93.33%
Mitral Regurgitation	Severe	14	15.56%
	Moderate	19	21.11%
	Mild	36	40.00%
	Normal	21	23.33%
Mitral Stenosis	Severe	47	52.22%
	Moderate	25	27.78%
	Mild	14	15.56%
	Normal	4	4.44%
Aortic Stenosis	Severe	11	12.22%
	Moderate	7	7.78%
	Mild	17	18.89%
	Normal	55	61.11%
Aortic Regurgitation	Severe	2	2.22%
	Moderate	18	20.00%
	Mild	20	22.22%
	Normal	50	55.56%
Tricuspid Stenosis	Moderate	1	1.11%
	Mild	4	4.44%
	Normal	85	94.44%
Tricuspid Regurgitation	Severe	20	22.22%
	Moderate	41	45.56%
	Mild	28	31.11%
	Normal	1	1.11%

## References

[1] Karthikeyan G (2017). Rheumatic heart disease in India: declining, but not fast enough. Natl Med J India.

[2] Harikrishnan S, Bahl A, Roy A (2022). Clinical profile and 90 day outcomes of 10 851 heart failure patients across India: National Heart Failure Registry. ESC Heart Fail.

[3] Hmadeh S, Trimaille A, Matsushita K (2024). Human aortic stenotic valve-derived extracellular vesicles induce endothelial dysfunction and thrombogenicity through AT1R/NADPH oxidases/SGLT2 pro-oxidant pathway. JACC Basic Transl Sci.

[4] Pabel S, Hamdani N, Luedde M, Sossalla S (2021). SGLT2 inhibitors and their mode of action in heart failure: has the mystery been unravelled?. Curr Heart Fail Rep.

[5] McMurray JJ, Solomon SD, Inzucchi SE (2019). Dapagliflozin in patients with heart failure and reduced ejection fraction. N Engl J Med.

[6] Packer M, Anker SD, Butler J (2020). Cardiovascular and renal outcomes with empagliflozin in heart failure. N Engl J Med.

[7] Anker SD, Butler J, Filippatos G (2021). Empagliflozin in heart failure with a preserved ejection fraction. N Engl J Med.

[8] White PD, Myers MM (1921). The classification of cardiac diagnosis. JAMA.

[9] Reményi B, Wilson N, Steer A (2012). World Heart Federation criteria for echocardiographic diagnosis of rheumatic heart disease: an evidence-based guideline. Nat Rev Cardiol.

[10] Zoghbi WA, Adams D, Bonow RO (2017). Recommendations for noninvasive evaluation of native valvular regurgitation: a report from the American Society of Echocardiography developed in collaboration with the Society for Cardiovascular Magnetic Resonance. J Am Soc Echocardiogr.

[11] Nunes MC, Tan TC, Elmariah S (2014). The echo score revisited: impact of incorporating commissural morphology and leaflet displacement to the prediction of outcome for patients undergoing percutaneous mitral valvuloplasty. Circulation.

[12] Negi PC, Kandoria A, Asotra S (2020). Gender differences in the epidemiology of rheumatic fever/rheumatic heart disease (RF/RHD) patient population of hill state of northern India; 9 years prospective hospital based, HP-RHD registry. Indian Heart J.

[13] Butt JH, Nicolau JC, Verma S (2021). Efficacy and safety of dapagliflozin according to aetiology in heart failure with reduced ejection fraction: insights from the DAPA-HF trial. Eur J Heart Fail.

[14] Shenthar J (2022). Management of atrial fibrillation in rheumatic heart disease. Heart Rhythm O2.

[15] Das PP, Malviya A, Mishra A, Buragohain D, Shashank, Goswami D, Preethi (2023). Cardiovascular outcome with SGLT2 inhibitor in rheumatic heart disease with left ventricular systolic dysfunction: an observational study. Heart Fail J India.

